# The prevalence and risk factors of allergic and respiratory symptoms in a regional cohort of extremely low birth weight children (<1000 g)

**DOI:** 10.1186/1824-7288-39-4

**Published:** 2013-01-18

**Authors:** Przemko Kwinta, Grzegorz Lis, Malgorzata Klimek, Andrzej Grudzien, Tomasz Tomasik, Karolina Poplawska, Jacek Jozef Pietrzyk

**Affiliations:** 1 Department of Pediatrics, Jagiellonian University Medical College, Cracow, 30-663, Poland

**Keywords:** Prematurity, Follow-up, Spirometry, IgE, FeNO, Skin prick tests

## Abstract

**Background:**

Children who were <1000 g (ELBW extremely low birth weight) at birth more frequently present with wheezing which is the most common reason that pediatric consultation is sought. Therefore asthma is diagnosed very often. However is the asthma that is diagnosed in ELBW subjects atopic in origin, or is there a different etiology?

**Aim:**

To determine if ELBW infants are at higher risk for the development of allergic and respiratory symptoms and to establish if there were any specific risk factors for these symptoms.

**Methods:**

81 children born with a mean birthweight of 845 g (91% of available cohort) were evaluated at the mean age 6.7 years. The control group included 40 full-term children. The children were examined for clinical signs of allergy, and were subjected to the following tests: serum total IgE, skin prick tests (SPT), exhaled nitric oxide measurement (FeNO) and spirometry.

**Results:**

ELBW children had wheezing episodes more often (64% vs. 25%; OR (odds ratio): 5.38; 95% CI (confidence interval): 2.14-13.8) and were diagnosed more frequently with asthma (32% vs. 7.5%; OR: 5.83, 95% CI: 1.52-26) than their term born peers. The most important risk factors for wheezing persistence were hospitalization and wheezing episodes in first 24 months of life. Mean serum tIgE level (geometric mean: 32+/−4 vs. 56+/−4 kU/L; p=0.002) was higher and the number of children with positive results of tIgE level (12% vs. 32%; p=0.02) were more frequent in the control group. Children from the control group also more frequently had SPT, however this data was not statistically significant (11% vs. 24%; p=0.09). All of the ELBW had normal FeNO level (<=20 ppb), but 5 children from the control group had abnormal results (p=0.02). There was no difference between the groups in the occurrence of allergic symptoms.

**Conclusion:**

ELBW children have more frequent respiratory, but not allergic problems at the age of 6–7 years compared to children born at term. The need for rehospitalization in the first 2 years of life, was a more important risk factor of future respiratory problems at the age of 7 than perinatal factors, the diagnosis of bronchopulmonary dysplasia or allergy.

## Introduction

Despite improvements in perinatal care, the incidence of late complications of prematurity is not decreasing. On the other hand what is increasing is the survival of ELBW infants [1] which due to innovations in neonatology/perinatology in the past years such as less invasive treatment strategies aimed at the restriction of excessive oxygen and ventilation, a decrease in postnatal infections and the improvement of nutrition [2] are a completely different population from the ELBW infants of 30 years ago and in consequence require ongoing follow-up and analysis. ELBW infants are also a very distinct population that usually have the most severe complications of prematurity (such as respiratory distress syndrome, patent ductus arteriosus, intraventricular hemorrhage, sepsis and necrotizing enterocolitis), which unfortunately continue to cause health problems later in life. The morbidity and mortality of these complications is highest in infants born at the threshold of viability [3]. In this study we decided to focus on respiratory problems because they contribute significantly to the morbidity of prematurely born children and usually persist at least until school age. Furthermore, it is very likely, that some patients who go on to have respiratory diseases in adulthood have a history of premature birth [4,5]. Children who were <1501 g at birth also more frequently present with wheezing [6] which has been shown to be the most common reason that parents seek pediatric consultation. Therefore asthma is diagnosed very often, particularly in children born prematurely and in consequence they are often treated with antiallergic and antiasthmatic medications (inhaled or nasal steroids, antihistamines, leukotriene antagonists).

However is the asthma that is diagnosed in ELBW subjects atopic in origin, or does it have a different etiology altogether? Baraldi and colleagues [7] recently showed that nitric oxide fraction in exhaled air (FeNO) values which are an indirect marker of eosinophilic airway inflammation were lower in preterm children than in those with asthma and even as much as four times lower in children with BPD. There are also investigations proving that prematurity reduces the long-term risk of atopy [8-10], and the occurrence of diseases such as allergic rhinitis, eczema and atopic asthma. We will try to cover these and many other issues in this study which deals with the the respiratory and allergic outcomes of babies born with a gestational age <30 weeks and birth weight <1000 g (ELBW) compared to term-born children at the age of 6–7 years. Most studies related to this subject up to date have included VLBW subjects, so this study is a rare opportunity to see how these problems evolve in the population of ELBW children.

## Material and methods

A cross-sectional observational study was conducted in the Follow-up Pediatric Department of the Polish-American Children’s Hospital between August 1, 2009 and October 31, 2010.

From the 1 of September 2002 to the 31 of August 2004 one hundred and sixty-nine newborns with birth weights from 500 to 1000 g were born alive in the south-east district of Poland (Małopolska region). All children were hospitalized in three tertiary care Neonatal Intensive Care Units (NICU) in south-east Poland. Ninety-one infants were discharged home from the NICU. The children who were still alive at the age of 6–7 years were invited to participate in the follow-up study (n=89). Neonatal data used for the study was recorded daily during their hospitalization in the NICU in a prospective manner and stored in computer databases. For the purpose of the study the following data was extracted from the original databases: sex, birthweight, gestational age, intrauterine growth parameters, Apgar score, incidence of preeclampsia, preterm rupture of membranes, *chorionamnionitis*, presence of respiratory distress syndrome (RDS), need for mechanical ventilation, surfactant administration, use of ibuprofen for patent ductus arteriosus (PDA), PDA ligation, early- and late-onset septic episodes, prevalence of intraventricular hemorrhage (IVH), periventricular leukomalacia (PVL), bronchopulmonary dysplasia (BPD) - defined as at least 28 days of oxygen therapy, moderate and severe BPD defined as oxygen therapy at 36 weeks post menstrual age (PMA), weight gain during NICU stay and length of hospitalization.

The control group included age-matched children from one general practitioners (GP) office. 42 children in total fulfilled the qualifying criteria of the study, the majority of parents agreed to let their children participate in the study (n=40), only 2 declined.

The study was approved by the Ethical Committee for Clinical Investigations of Collegium Medicum, Jagiellonian University.

After signing an informed consent, parents were asked to complete two questionnaires: 1/ including demographic variables, family characteristics (education level, health status, place of residence (city/rural area), parental atopy status), nutrition during the first year of life (breast-feeding/formula feeding), environmental risk factors of allergy (parental smoking habits, siblings, day care attendance, home pets), history of treatment and hospitalizations due to respiratory problems; 2/ validated, standardized ISAAC (International Study of Asthma and Allergies in Childhood) questionnaire assessing allergic disorders [11]. All questions were verified by a physician during face-to-face discussion. Afterwards, all children were examined by an investigator for the presence of atopic eczema, (rhino)conjunctivitis, wheezing, and other clinical signs of allergy.

### Laboratory evaluation

After the examination, a venous blood sample (3 ml) was taken for the assessment of absolute eosinophil count and serum total IgE (tIgE). tIgE levels were measured using the immunofluorometric method (ImmunoCAP, Phadia AB, Sweden) with an assay sensitivity range of 2–5000 kU/L. Serum tIgE levels above the upper limit for age of the patient were recognized as a positive result. Absolute eosinophil counts were calculated according to standard hospital laboratory methods.

During the same visit skin prick tests (SPT) were performed in a typical manner, for the following 10 allergens (Allergopharma, Germany): indoor allergens - house dust mites (*D. pteronyssimus*, *D. farine*), dog, cat, molds (*Alternaria, Aspergillus, Cladosporium*), outdoor allergens – grasses, trees, weeds. A reaction to an allergen was regarded positive if the mean wheal diameter was at least 3 mm. Atopy was defined as a positive SPT to at least one of the aeroallergens.

### Exhaled nitric oxide

Exhaled nitric oxide (FeNO) was measured in concordance with published standards [12], using an electrochemical hand-held device - NIOX MINO® (Airway Inflammation Monitor /NIOX MINO/, Aerocrine AB, Solna, Sweden), following the producers instructions, with exhaled air flows equal to 50±5 ml/s. Measurements of FeNO were performed prior to all other study procedures.

FeNO results were evaluated according to the guidelines by Taylor et al. in which - levels equal or below 20 ppb were regarded as normal [13].

### Lung function

Spirometry was performed using a Lungtest 1000 spirometer with a pneumotachometer-based system (MES, Kraków, Poland). The forced vital capacity (FVC), forced expiratory volume in one second (FEV_1_), ratio of FEV_1_/FVC and the forced expiratory flow at 50% of FVC (FEF_50_) was measured before and after the inhalation of 400 ug of salbutamol, according to the recommendations of ATS/ERS [14]. Results are presented as percent of predicted values with reference values of Zapletal *et al.*[15]. Bronchodilator response was estimated and expressed as the percentage of FEV_1_, FEF_50_ increase after salbutamol inhalation (Δ%FEV_1_, Δ%FEF_50_). A Δ%FEV1 of more than >12% was considered a significant bronchodilator response.

### Outcome variables

The primary outcomes at the age of 7 years were the presence of respiratory and allergic problems defined as a positive response to the ISAAC questionnaire pertaining to : 1) wheezing in the last year, 2) sneezing or a runny or blocked nose with itchy-watery eyes without cold or flu in the last year (rhinoconjuctivitis in the last year), 3) itchy rash in the last year with any flexural involvement or located around the neck, ears or eyes (eczema symptoms in the last year), 4) any wheezing episode in the past (wheeze ever), 5) the diagnosis of asthma (asthma) 6) hay fever/allergic rhinitis (allergic rhinitis), or 7) eczema by a doctor in the past (eczema). Moreover, we also asked: Has your child ever been hospitalized due to respiratory problems? (hospitalizations ever), Was your child hospitalized due to respiratory problems in the its first 24 months of life? (hospitalization in the first 24 months of life), Was your child hospitalized for respiratory problems in the last year? (hospitalizations in the last year), Is your child taking any respiratory or allergic medications? (current medications).

Secondary outcome variables were positive results of serum tIgE or SPT and the subject’s respiratory status determined by spirometry results and FeNO.

### Statistical methods

In order to draw comparisons between the groups, the following tests were utilized as was deemed appropriate: Student’s *t*-test, ANOVA, Kruskal-Wallis test, chi square test or Fisher’s exact test. Odds ratio (OR) and 95% confidence interval (CI) were calculated for the risk of respiratory and atopic problems.

Statistical significance was defined for two sided test at the p=0.05 level. Data was analyzed using SAS Software (2006 by SAS Institute Inc., Cary, NC, USA).

Sample size estimations were based on the assumption that the follow-up rate among ELBW children would be as high as 90% (80 ELBW children). The frequency of primary endpoints was adopted on the basis of an ISAAC study, in which the incidence of wheeze in the last year, rhinoconjuctivitis in the last year, and eczema symptoms in the last year ranged from 15 to 30%. Assuming that the risk of type I error equaled to 5% (two sided test) and the control group would include 40 children, a study with the power of 80% might demonstrate a 15% difference in the incidence of primary endpoints between ELBW and control group subjects. Sample size calculation was performed using PS Power and Sample Size Calculations software (Version 2.1.30, February 2003, http://www.mc.vanderbilt.edu/prevmed/ps/index.htm)

## Results

Eighty-one children born as ELBW infants (91% of the available cohort) with a mean birthweight of 849 g (SD: 131 g) and a mean gestational age of 27.2 weeks (SD: 2.1 weeks) were evaluated at a mean age of 6.7 years (SD: 0.4). The control group included 40 full-term children. The comparison of selected demographic variables between the studied groups is shown in Table 1. Vaginal delivery was more frequent in the control group. ELBW children were more frequently small for gestational age than children from the control group (about 30% of them). The groups were similar with respect to age and gender.

**Table 1:** **Comparison of selected demographic and clinical variables between ELBW newborns and the control group**^**a**^

	**ELBW group (n=81)**	**Control group (n=40)**	**p value**
Birth weight (g), mean (SD)	845 (130)	3554 (512)	<0.001^b^
Gestational age (weeks), mean (SD)	27.2 (2.1)	39.9 (1.4)	<0.001^b^
Female	52 (64)	21 (53)	0.15^c^
Vaginal delivery	17 (21)	34 (85)	<0.001^c^
Multiple pregnancy	10 (12)	0 (0)	<0.001^c^
Small for gestational age	24 (30)	2 (5)	0.003^c^
5th min. Apgar score, Me; (25th -75th percentile)	6 (5–7)	10 (9–10)	<0.001^d^
Siblings	47 (58%)	27 (67.5%)	0.33^c^
School attendance	71 (88%)	40 (100%)	0.03^c^
Paternal history of atopy	14 (17%)	3 (7.5%)	0.17^c^
Pets at home	32 (39.5%)	13 (32.5%)	0.7^c^
Passive smoking			
None	64	32	
History	9	1	0.16^c^
Current	8	7	
Place of residence			
City area	31	18	0.56^c^
Rural area	50	22	

^a^expressed as a number (percentage) of patients unless otherwise indicated.

p value for ANOVA^b^, chi square test^c^, and Kruskal-Wallis test^d^.

ELBW – extremely low birth weight.

The comparison of factors which could have an impact on the respiratory system in the studied groups is shown in the Table 1. There were no differences in number of siblings, family history of atopy, history of passive smoking, presence of any pets at home and place of residence. Breast feeding after 1 month of life was less common in the ELBW group, in addition 12% of them did not attend school.

**Figure 1:**
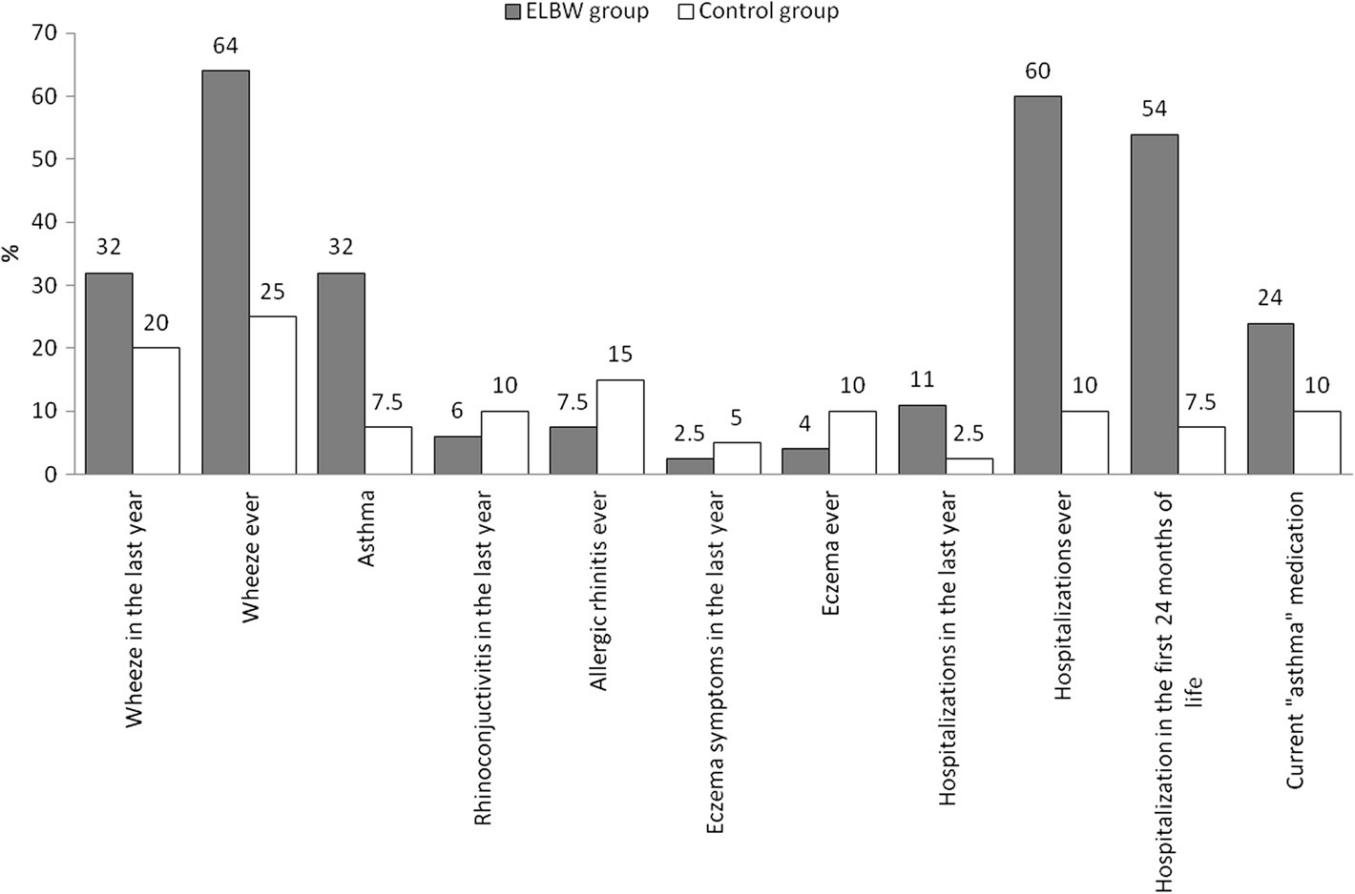
Comparison of the presence of respiratory and atopic problems between the groups at 6–7 years of age.

The results of the questionnaire relating to respiratory and atopic problems are presented in Figure 1. Wheeze ever was reported more frequently in the ELBW group (64% of the ELBW vs 25% of control group, OR 5.38, 95% CI: 2.14-13.8), although in the last year that difference had decreased and was no longer statistically significant (32% vs 20%). Asthma was diagnosed in a large number of ELBW children (32% of ELBW vs 7.5% of the control group, OR 5.83, 95% CI: 1.52-26). The majority of ELBW children required hospitalization due to respiratory problems (hospitalization ever: 60% of ELBW vs 10% of control, OR 13.8, 95% CI: 4.13-51). The hospitalization rate had decreased in the last year, nevertheless it was still four times higher in ELBW than in the control group. The current use of antiallergic and antiasthmatic medications (inhaled or nasal steroids, antihistamines, leukotriene antagonists) was 2 times higher in the ELBW group in comparison to the control group (although this difference was not statistically significant). There was no difference between the groups in the occurrence of other allergic diseases or symptoms such as: allergic rhinitis or eczema ever, rhinoconjuctivitis, eczema symptoms in the last year (all symptoms were less frequent in the ELBW group).

The results of laboratory tests in the studied groups are presented in Table 2.

**Table 2:** Laboratory tests results in the studied groups

	**ELBW group**	**Control group**	**p value**
	**N=22**	**N=20**	
**Spirometry and bronchial responsiveness (expressed as mean ± SD)**			
FEV_1_ (%)	81.3±13	95.8±8	<0.001^a^
FVC (%)	79±13	89±7	<0.001^a^
MEF_50_ (%)	62±19	87±26	<0.001^a^
Δ%FEV_1_ (%)	6.8±12.3	3.6±5.9	0.3^a^
Δ%MEF_50_ (%)	31±32	19±17	0.15^a^
FEV_1_ < 80% predicted value	10 (45%)	0	0.001^b^
Δ%FEV_1_ >12%	4 (18%)	1 (5%)	0.3^b^
**FeNO** (ppb)			
Median (25^th^-75^th^ percentile)	8 (8–13)	10 (8–14)	0.2^c^
Normal (≤20 ppb)	22	15	0.02^b^
Intermediate (20–35 ppb)	0	3	
High (>35 ppb)	0	2	
	**N=81**	**N=40**	
**serum tIgE** (kU/L)			
Geometric mean (± SD)	32±4	56±4	0.002^a^
High (>upper limit for age)	10 (12%)	12 (32%)	0.02^b^
**Others**			
Absolute eosinophil count per uL)	265±202	225±185	0.3^a^
Positive SPT	8 (11%)	9 (24%)	0.09^b^

p value for ANOVA^a^, chi square test^b^, and Kruskal-Wallis test.

ELBW – extremely low birth weight.

SPT – skin prick test.

**Figure 2:**
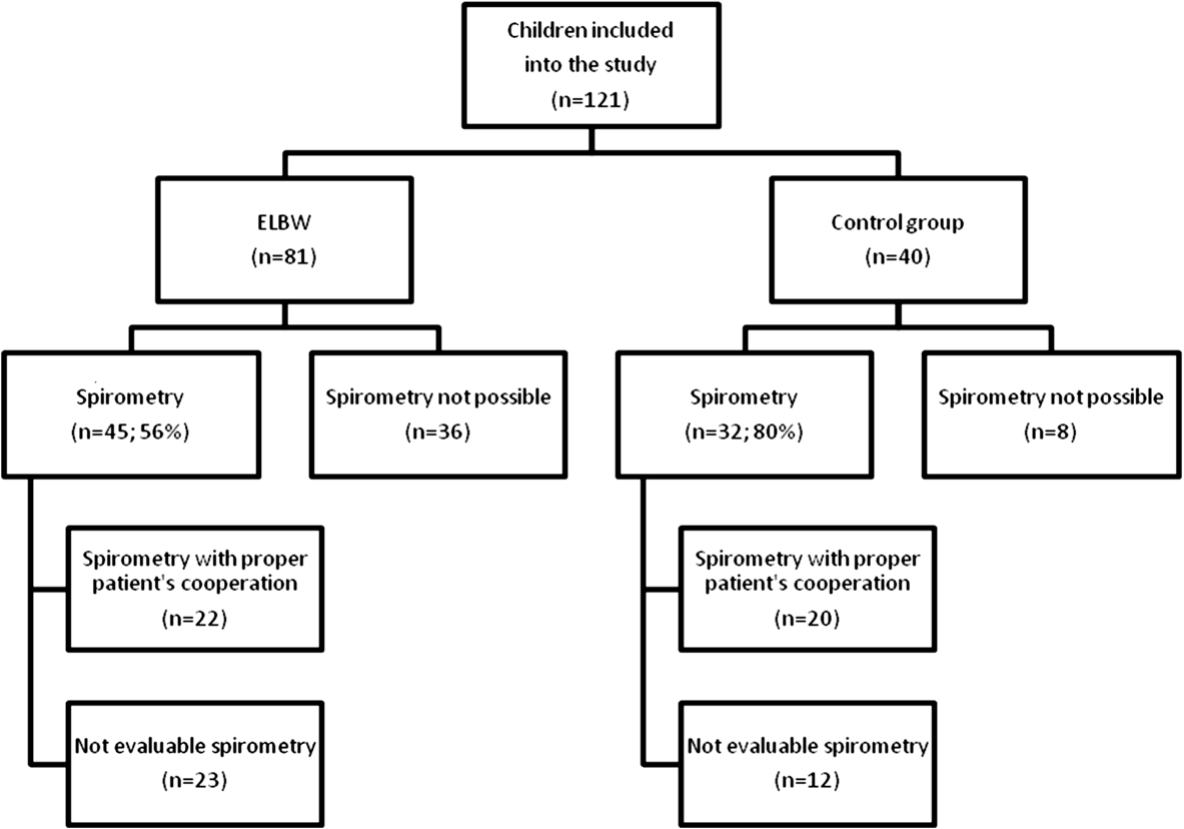
Study flow-chart.

**Figure 3:**
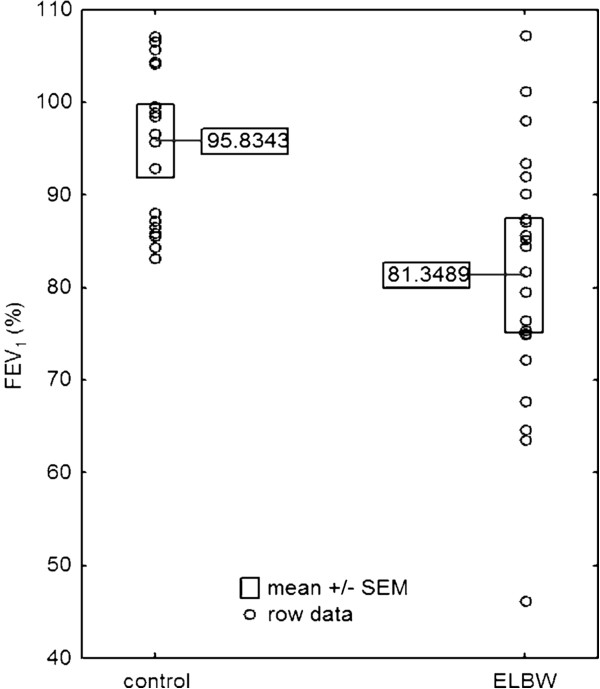
Comparison of forced expiratory volume measurements between the group of extremely low birthweight children and the group of full-term peers.

Spirometry was performed in 56% (45/81) of ELBW children and in 80% (32/40) of children in the control group (Figure 2). In 44% of ELBW children it was not possible to perform the spirometry because of their neurological complications or lack of child’s cooperation. A quarter of the ELBW group (22/81) and half of the control group (20/40) were able to perform acceptable and repeatable spirometry that conformed to ATS/ERS standards for this age group of children. The ELBW children had a significantly lower FEV1 (Figure 3), FVC and FEF50 compared to control subjects. FEV1 lower than 80% of the predicted value was observed in up to half of the ELBW group (10/22) and not in the control group. The values of Δ%FEV1 and Δ%FEF50 were similar in both groups. A significant bronchodilator response was observed in 4/22 of ELBW and in 1/20 of control group subjects (difference between groups was not statistically significant).

### FeNO

Appropriate maneuvers for FeNO measurements were obtained in 22 ELBW children and in 20 of the controls. All of the ELBW participants had normal FeNO levels (≤20 ppb). Five children from the control group had abnormal results: 3 had results ranging from 20–35 ppb and 2 above 35 ppb.

### Atopy

Serum tIgE (geometric mean) and the number of children with positive results of tIgE levels were more frequent in the control group. Children from the control group also more frequently had positive SPT, however this data was not statistically significant. There was no difference in absolute eosinophil counts between studied groups.

### ELBW children with vs. without wheeze

Comparison of selected factors in ELBW children with or without wheezing in the last year is presented in Table 3. There were no differences between groups due to: birth weight, gestational age, gender, length of mechanical ventilation, length of oxygen therapy and indicators of bronchopulmonary dysplasia – oxygen therapy at least 28 days and oxygen therapy at 36 weeks postmenstrual age. The group of ELBW with wheezing in the last year was characterized by more frequent wheezing in the first 24 months of life and more hospitalizations due to respiratory tract infections in that time period. There was no difference regarding: family history of atopy, passive smoking, pets at home, tIgE and positive SPT. The difference in the frequency of hospitalizations in last year, as well as current medication use - were more frequent in the group with wheeze in the last year. The spirometry revealed no difference in FEV_1_ between both groups, but a higher Δ%FEV_1_ in the group with wheeze in the last year. Only in that group half of the children had an increase in Δ%FEV_1_ greater than 12%.

**Table 3:** Comparison of selected factors between the group of ELBW infants with and without wheeze in the last year

	**Without wheeze in last year (n=55)**	**Wheeze in the last year (n=26)**	**p value**
**Perinatal factors**			
Birth weight (g), mean (SD)	853±139	830±159	0.8^a^
Gestational age (weeks), mean (SD)	27.3±2.1	27.3±2.6	0.9^a^
Female	37 (67%)	15 (58%)	0.5^b^
Length of mechanical ventilation (Me; 25-75^th^ percentile) (days)	17 (2–48)	24 (6–39)	0.8^c^
Length of oxygen therapy (Me; 25-75^th^ percentile) (days)	44 (13–77)	55 (34–74)	0.9^c^
Oxygen therapy at least 28 days	34 (62%)	19 (73%)	0.5^b^
Oxygen therapy at the 36 PMA	21 (38%)	10 (38%)	1.0^b^
**Infancy**			
Wheeze in the first 24 months of life	29 (53%)	21 (81%)	0.03^b^
Hospitalization in the first 24 months of life	25 (45%)	19 (73%)	0.03^b^
Immunoprophylaxis with palimivizumab	8 (15%)	2 (8%)	0.5^b^
**Atopy**			
Parental history of atopy	9 (17%)	5 (22%)	0.8^b^
Passive smoking	4 (7%)	4 (15%)	0.26^b^
Pets at home	21 (40%)	11 (48%)	0.7^b^
serum tIgE (Me; 25-75^th^ percentile)	22 (12–68)	34 (12–73)	0.3^c^
Absoulte eosinophil count (eos/uL	230±137	338±275	0.03^a^
Positive SPT	4 (7%)	4 (15%)	0.26^b^
**Recent status**			
Hospitalization in the last year	2 (4%)	12 (46%)	<0.01^b^
Current medications	11 (20%)	15 (58%)	0.002^b^
FEV_1_ (%)	80±13	83±16	0.61^a^
Δ%FEV_1_ (%)	1±7	16±13	0.002^a^
Δ%FEV_1_ >12%	0/14	4/8 (50%)	0.01^b^

p value for ANOVA^a^, chi square test^b^, and Kruskal-Wallis test.

ELBW – extremely low birth weight.

### AGA vs SGA infants

Furthermore we compared the incidence of complications in ELBW infants based on intrauterine growth parameters, dividing the subjects into two groups: small for gestational age (SGA) and appropriate for gestational age (AGA). No significant differences were found in the incidence of respiratory symptoms such as wheeze ever or wheeze in the last year in SGA compared to AGA subjects. The diagnosis of asthma was less common in SGA subjects (25% compared to 35%) although this result was not statistically significant.The occurrence of allergic symptoms in both groups were comparable (data available on request). According to laboratory tests we found a higher incidence of positive SPT and positive results of tIgE levels in SGA compared to AGA participants although this was not statistically significant as well (p=0.1).

## Discussion

In this study we presented respiratory and allergic problems in the geographically based cohort of ELBW children at the mean age of 6.7 years.

In our opinion, the study has significant value because: 1/ the study group included almost all newborns from the whole Malopolska region born in a period of 2 years that reached the age of 6–7 years. The data in our multi-center study comes from all the tertiary referral centers from the Malopolska region. It is a complete group of patients with a high percentage of observation (91%). 2/ The assessment of the past and current health status of the child was based on a validated, standardized ISAAC questionnaire and all responses were verified by a physician. Moreover, all children were examined by an investigator for the presence of symptoms characteristic of eczema, rhinoconjunctivitis, wheezing, and other clinical signs of allergic disorders. 3/ The current study limited the study group to ELBW children, most of the previous studies included VLBW infants. 4/ Lung function was evaluated by spirometry according to the recommendations of ATS/ERS published in 2005 [14]. 5/ In addition, FeNO measurements which are a marker of eosinophilic airway inflammation were done as well.

One of the major limitations of the study was the fact that the spirometry results suitable for evaluation were completed by only a quarter of the ELBW participants (22/81) and half of the control group (20/40). Considering the fact that VLBW infants are at a greater risk of neurodevelopmental delay, cerebral palsy, hearing impairment and cognitive and emotional problems later in life [16], cooperation in this group of children is a challenge and the achievement of reproducible spirometric efforts is extremely difficult. Not to mention the fact that this task can be sometimes difficult among term born children as well.

One of the major results of this study was the observation that among ELBW subjects, the children with wheezing episodes in the last year more often had the need for hospitalization in the first 2 years of life compared to other (non-wheeze in the last year) ELBW children. Surprisingly we found no difference in birth weight, gestational age, length of mechanical ventilation, oxygen therapy in the first 28 days of life or at 36 weeks PMA between ELBW children with and without wheeze in last year. There was no significant difference in family history of atopy, pets at home between ELBW children with and without wheeze in last year. Furthermore those children were not exposed more often to passive smoking. Interestingly enough, positive SPT were not more frequent and serum tIgE levels were not greater in the ELBW population with wheeze in last year. Thus there was no evidence of atopy contributing to persistent respiratory problems in those children. The baseline FEV_1_ values were similar, but %FEV_1_ values were greater in the ELBW children with wheeze in last year and in half of them there was a significant bronchodilator response.

Our results suggest that symptoms of asthma observed in the ELBW population correlated more strongly with respiratory morbidity in infancy rather than perinatal problems or atopic factors. We have shown that the need for re-hospitalization in the first 2 years of life, was a more important risk factor of respiratory problems at the age of 7 than perinatal factors or the diagnosis of bronchopulmonary dysplasia. Furman *et al.*[17] who followed 98 out of 124 VLBW infants with BPD up to the age of 2 years also showed that the severity of BPD correlated with the duration of neonatal hospital stay and total hospital stay during the first 2 years of life. A similar finding was also mentioned in another study by Ballow et al. [18] which followed 43 VLBW preterm infants until 10 months chronological age and found that they had a considerably higher incidence of infections (especially lower respiratory tract infections) than term infants, what’s more none of them turned out to be bacterial infections. Among lower respiratory tract infections RSV has been shown to be the most common cause in high risk infants such as prematurely born infants [19] in addition it has been shown preterm infants are more likely to have a more severe clinical course of the disease than term infants (more prolonged hospital stays, requiring oxygen for longer periods of time, more frequent usage of nasal continuous positive airway pressure, CPAP, or mechanical ventilation) [20].

In light of these facts it is highly probable that the increased hospitalisation rate in ELBW infants is not only due to the immaturity of the lungs, not fully recovered from the morbidity of the perinatal period but also due to the damage to the lungs that is incurred with each subsequent infection and also through the use of respiratory support (volutrauma, barotrauma, oxygen toxicity) causing a state of chronic inflammation and lung injury.

Our study confirmed previous observations that ELBW children had more frequent respiratory problems at the age of 6–7 years compared to children born at term. In ELBW children, wheeze in the last year or ever in the past and an established diagnosis of asthma were observed more frequently in these subjects than in their peers. The need for hospitalization due to respiratory disease at any time was significantly higher in the ELBW population. This tendency was also present in the last year, however it did not have full statistic significance. However the results of the pulmonary function tests were significantly poorer in ELBW subjects than in controls. This explains why the use of medications – first and foremost- inhaled steroids were more common in the ELBW group. The need for hospital admissions also suggested that the severity of respiratory disease was greater in ELBW children. Fortunately there has been a decrease in the need for hospitalization in the last year.

Similar observations have been noted in others studies. McLeod *et al.* studied a group of very low birth weight (VLBW) children who were evaluated at the age of 8–9 years and reported that they used inhaled drugs and were admitted to hospital more often than their classroom peers [21]. Siltanen *et al.* reported an increased prevalence of wheezing in preterm infants (43%) at the age of 10 years compared to term-born subjects (17%) [22]. A reduction in the number of hospital admissions after the second year of life, including children with BPD, was reported in another study [23].

Martinez *et al.* reported that children who started wheezing early in life and continued to wheeze at the age of six years were more likely to have a family history of asthma and elevated serum tIgE. However, the gestational age of the subjects was not revealed in that study [24]. In our population the ELBW children did not present with: allergic rhinitis, rhinoconjunctivitis or eczema more frequently than their term born peers. Bűhrer *et al.* also showed a decreased prevalence of atopic eczema in VLBW infants compared to term and near-term infants in the first year of life suggesting that early antigen exposure in VLBW infants could lead to tolerance and a decreased risk of sensitization [25].

Risk factors of allergy development such as: family history of atopy, exposure to tobacco smoke, contact with animals (pets at home, indoor allergens), place of residence, presence of siblings at home were similar in both groups. The only differentiating factor was duration of breast feeding, which in ELBW children frequently lasted less than one month, meanwhile breast feeding lasting more than 4 months has been proven to reduce the risk of asthma at the age of 6 years [26].

Asthma was diagnosed more often in the ELBW group (32%) than in the control group (7.5%). In other reports, asthma was also diagnosed more frequently in ELBW children (24.7%) than in controls (13.9%) at the age of 8–9 years [27] and 28% vs 14% at the age of 10 years, respectively [28]. Large-scale analysis conducted by Brooks *et al.* confirmed a strong independent association between low birth weight and asthma, diagnosed by a physician at the age of 3 years [29]. It is important to note, that the prevalence of asthma in our controls corresponded with the results of the ISAAC survey of 6–7 year old children in a proper geographic region [11].

Siltanen *et al.* reported that atopy was more frequent in term than in the ELBW infants, and reduced pulmonary function in that group was not related to atopy [9,10,22]. Mieskonen *et al.* showed that atopy was less common in VLBW children with BPD than without BPD, furthermore atopic children had higher birth weights, a shorter need for ventilator and oxygen therapy than non-atopic children born prematurely [30]. In our study the serum tIgE was higher and its level more frequently above the upper limits for age in the control group. The SPT were also more frequently positive in the control group, but the difference was not statistically significant. Thus, we can conclude that symptoms of asthma in our cohort of ELBW children were not associated with atopy.

In our study FEV1 below 80% of predicted value was observed in the nearly half of ELBW children and not in the control subjects. Similar observations were made in children with symptoms suggestive of asthma and bronchopulmonary dysplasia [31].

Doyle *et al.* reported that all respiratory function variables reflecting airflow were diminished in the ELBW group compared to term-born controls at the age of 8–9 years (reduction in FEV_1_ below 75% was observed in 19.7% ELBW children vs 2.4% in control subjects) [27]. Mieskonen *et al.* reported that BPD and non-BPD VLBW children had significantly lower FVC, FEV_1_, FEF_50_ than controls, but the BPD VLBW children had significantly lower values than non-BPD VLBW at 8 years of age [30]. Mai *et al.* evaluated VLBW compared to term children at the age of 12 years, a history of asthma was more frequently noted in VLBW children and the only significant risk factor was prolonged oxygen therapy. Spirometric values were similar among VLBW and term children [8]. The improvement in spirometry results (FEV_1_) obtained in VLBW subjects between 6 and 12 years of age indicated that there was an acceleration of the lung development, although they did not catch up with their peers in weight and height [8,28].

Eosinophilic airway inflammation was assessed by measurement of FeNO. Atopy and atopic asthma leads to the increase in FeNO levels [30,32]. Diminished levels of FeNO were observed in children with virus-associated acute wheezy bronchitis [33]. The level of FeNO correlated with BAL fluid or sputum eosinophil percentage, but not with other inflammatory cells, and is considered to be a marker of eosinophilic inflammation [34,35]. A relationship between FeNO and blood eosinophils was also confirmed [32]. In our study, the FeNO levels were not higher in the ELBW population (particularly not greater than 20 ppb), which can indicate the absence of an eosinophilic inflammatory process in their respiratory tract. However, we could not exclude the effect of inhaled steroids on FeNO levels in some patients. Glucocorticosteroids decrease FeNO in asthmatic, but not healthy subjects [31]. Baraldi et al. showed that FeNO was significantly lower in VLBW children with BPD than in VLBW without BPD (however, all results were below 11 ppb) [7]. Mieskonen et al. found no significant differences in FeNO levels between BPD and non-BPD non-atopic subjects [30]. Both authors revealed no difference in FeNO between VLBW children and healthy controls. The atopic VLBW children had significantly higher FeNO levels (mean 14.8 ppb) than non-atopic prematurely born subjects (mean 6.3 ppb), although it was not significantly higher than healthy term controls (mean 6.4 ppb) [30]. These authors found that flow limitation was not associated with increased NO production and lack of reversibility of airflow limitation and low FeNO values suggested different than asthma mechanism of lower forced expiratory flows. We had similar observations.

## Conclusions

In summary we demonstrated that respiratory problems were more frequent in ELBW children at the age of 6–7 years compared to children born at term. ELBW children had more episodes of wheezing, were more frequently diagnosed with asthma and had decreased spirometry parameters compared to their peers. The need for hospital admissions due to respiratory problems was also greater in the ELBW population (although it had decreased in the last year) as was the use of medications, especially inhaled steroids.

However the occurrence of other allergic diseases such as allergic rhinitis, or eczema was not observed more frequently in the ELBW population, nor were symptoms of rhinoconjunctivitis, eczema in the last year. We also did not show an elevation of FeNO in ELBW children which can be assumed as a surrogate of eosinophilic airway inflammation. Mean serum tIgE levels were higher and the number of children with positive results of tIgE levels were more frequent in the control group. Children from the control group also more frequently had positive SPT, however this data was not statistically significant.

On the other hand we found that the need for re-hospitalization in the first 2 years of life was the most important risk factor for the occurrence of respiratory problems at the age of 6–7 years, even more relevant than perinatal factors and the diagnosis of bronchopulmunonary dysplasia. A lot of the studies mentioned in the discussion as well as our own results have shown a decreasing tendency of respiratory problems in ELBW children as they grow up. Longitudinal follow up studies are needed to determine whether this trend will continue but also whether these subjects will be more susceptible to developing pulmonary disease later in life such as COPD (Chronic obstructive pulmonary disease).

## Abbreviations

BPD: Bronchopulmonary dysplasia; CI: Confidence interval; COPD: Chronic obstructive pulmonary disease; ELBW: Extremely low birth weight; FEF_50_: Forced expiratory flow at 50% of FVC; FeNO: Exhaled nitric oxide measurement; FEV_1_: Forced expiratory volume in one second; FVC: Forced vital capacity; GP: General practitioner; ISAAC: International Study of Asthma and Allergies in Childhood; IUGR: Intrauterine growth retardation; IVH: Intraventricular hemorrhage; NICU: Neonatal intensive care unit; OR: Odds ratio; PDA: Patent ductus arteriosus; PMA: Post menstrual age; PVL: Periventricular leukomalacia; RDS: Respiratory distress syndrome; SPT: Skin prick test; tIgE level: Total Immunoglobulin E; VLBW: Very low birth weight.

## Competing interests

The authors declare that they have no competing interests.

## Authors’ contributions

PK-invented the concept of the study, recruited participants, analyzed and interpreted data, was involved in drafting the manuscript and revising it critically for important intellectual content. GL-carried out the spirometric studies but also took part in the analysis and interpretation of data. MK- recruited participants, physically examined the study participants for the presence of atopic eczema, (rhino)conjunctivitis, wheezing, and other clinical signs of allergy, carried out the questionnaires . AG- took part in the acquisition of data, helped to draft the manuscript. TT- took part in the acquisition of data, helped to draft the manuscript. KP- took part in the acquisition of data, helped to draft the manuscript. JJP- coinvented the concept of the study, recruited participants, analyzed and interpreted data, was involved in drafting the manuscript and revising it critically for important intellectual content. All authors read and approved the final manuscript.
